# Supplementary material for: MetaRibo-Seq measures translation in microbiomes
Source: Nat Commun. 2020 Jun 29;11:3268. doi: 10.1038/s41467-020-17081-z (PMC7324362; doi:10.1038/s41467-020-17081-z)
Supplement: Supplementary file 10 — Supplementary Data 7 [file 41467_2020_17081_MOESM10_ESM.zip › File2/Confidence_High_Taxonomy/357468_out.krona.html]

Javascript must be enabled to view this page.

members
magnitude
magnitudeUnassigned
count
unassigned
taxon
rank

357468\_out

3

2
3
superkingdom

phylum
976
3

class
3
200643

3
171549
order

family
171552
3

3
838
genus


SRS012849\_contig\_number\_18682SRS104327\_contig\_number\_7680SRS148253\_contig\_number\_12635
species
3
165179
